# Evaluation of the Performance of Nitrate Reductase Assay for Rapid Drug-susceptibility Testing of *Mycobacterium tuberculosis* in North India

**DOI:** 10.3329/jhpn.v29i1.7563

**Published:** 2011-02

**Authors:** Anamika Gupta, Malay Ranjan Sen, Tribhuban Mohan Mohapatra, Shampa Anupurba

**Affiliations:** **Department of Microbiology, Institute of Medical Sciences, Banaras Hindu University, Varanasi 221 005, India**

**Keywords:** Antitubercular agents, *Mycobacterium tuberculosis*, Drug resistance, Mutiple, Nitrate reductase assay, Tuberculosis, Multi-drug-resistant, India

## Abstract

The objective of the study was to evaluate the performance of nitrate reductase assay (NRA) as a rapid, reliable and inexpensive method for drug-susceptibility testing (DST) of *Mycobacterium tuberculosis* against first-line antitubercular drugs, such as rifampicin (RIF), isoniazid (INH), streptomycin (STR), and ethambutol (EMB). In total, 286 isolates were subjected to test by proportion method (PM) and NRA. By comparing the results of NRA with those of the gold standard PM, sensitivities and specificities were 98.4%, 97%, 88.5%, and 94.2% and 100%, 100%, 94%, and 99% for RIF, INH, STR, and EMB respectively. The positive predictive values were 100%, 100%, 95%, and 98% for RIF, INH, STR, and EMB respectively. The negative values were 99%, 98%, 87%, and 96% for RIF, INH, STR, and EMB respectively. The median time of obtaining results was shorter using NRA (10 days) compared to PM (28 days). An excellent agreement was observed between the two phenotypic tests with the κ values of 0.98, 0.97, 0.81, and 0.93 for RIF, INH, STR, and EMB respectively. The results demonstrated that NRA is suitable for the early determination of INH and RIF resistance and has the potential to be a useful tool for rapid drug-sensitivity test of *M. tuberculosis* in resource-constrained settings.

## INTRODUCTION

Tuberculosis (TB) remains the greatest cause of illness and death worldwide, especially in Asia and Africa, which have accounted for 55% and 31% of the cases respectively in 2007 (1).

The first five countries in terms of total numbers of TB cases in 2007 were India (2 million), China (1.3 million), Indonesia (0.53 million), Nigeria (0.46 million), and South Africa (0.46 million). Of the 9.27 million incidences of TB cases throughout the world in 2007, an estimated 1.37 million (15%) were HIV-positive; 79% of these HIV-positive cases were in the African region, and 11% were in the South-East Asia region. Developing countries account for 95% of active TB cases and deaths due to TB worldwide (2). Malnutrition and poor sanitary conditions contribute to the large-scale occurrence of the disease. The HIV pandemic has also played a role in enhancing the burden of the disease all over the world (3).

In developing countries, many national TB-control programmes have failed because of their low case-detection rates, and once a case is detected, cure may also be difficult because of poor case management and inadequate control of drug prescription (4). Furthermore, the most worrisome trend in recent years, which challenged the global prospects for TB control, is an increase in drug-resistant TB, particularly multidrug-resistant TB (MDR-TB), defined as resistance to at least rifampicin (RIF) and isoniazid (INH). Extensively drug-resistant TB (XDR-TB) occurs in MDR strains which are resistant to any fluoroquinolone and at least one of three injectable second-line aminoglycoside drugs, i.e. amikacin, kanamycin, or capreomycin. In response to the emergence of XDR-TB, the World Health Organization (WHO) is now calling for universal access to culture and DST by 2015 for all new pulmonary TB patients (5). According to the WHO, 500,000 new cases of MDR-TB occur globally every year, and MDR-TB has been reported in 2.9% and 15.3% of new and previously-treated cases respectively (6).

There is an urgent demand for early and proper detection of MDR and XDR-TB cases for the effective management and control of TB. Conventional method, i.e. proportion method (PM), is the gold standard for DST of *Mycobacterium tuberculosis* (MTB) but has some limitations, such as cumbersomeness and long-turnaround time (TAT). In recent years, a multitude of techniques for rapid DST has been designed and evaluated, such as colorimetric redox method (7), radiometric method BACTEC 460-TB (8), commercial MGIT, E-tests (9, 10), and molecular methods—Genotype MTBDR*plus* and INNO-LiPA (11, 12). Most of these have proved their reliability and accuracy but are limited to developed countries only because of high expenses while others, such as colorimetric redox methods or nitrate reductase assay, need more evaluation in the field.

NRA—also called Griess method (13)—was first des-cribed by Angeby *et al.* (14). NRA is based on the principle that MTB has the capability of reducing nitrate to nitrite. The presence of nitrite can be detected by addition of Griess reagent which changes the colour of the culture medium (15). Several studies evaluated the performance of NRA but only a few were in India (16-21). The present study was, therefore, conducted to evaluate the performance of NRA compared to the gold standard PM for DST of MTB. Susceptibility tests were performed for all four first-line antitubercular drugs, i.e. RIF, INH, streptomycin (STR), and ethambutol (EMB).

## MATERIALS AND METHODS

### Settings

*M. tuberculosis* strains were isolated from clinical sputum specimens collected from three different TB centres of Varanasi and from specimens submitted to the Department of Microbiology, Institute of Medical Sciences, Banaras Hindu University, India, for 22 months during January 2008–October 2009.

### Mycobacterial strains

In total, 286 strains of MTB were evaluated in the study. The isolates and sputum specimens were obtained from cases reported for pulmonary TB. H37Rv (ATCC 27294), and *M. intracellulare* (ATCC 13950) strains served as nitrate-positive and nitrate-negative controls respectively. A known MDR strain was also used as control. All the strains were first confirmed for their nitrate reductase activi-ty (22), which is part of the routine biochemical tests. The strains were confirmed to be MTB based on their growth rates, pigmentation, colony morphology, and biochemical tests, such as heat-stable catalase, niacin accumulation, and susceptibility to *p*-nitrobenzoic acid (PNB) (22). All the strains were sub-cultured in Lowenstein-Jensen (LJ) medium for four weeks before NRA was performed.

### Antitubercular drugs

INH, STR, RIF, and EMB were obtained in powder form from Sigma (St Louis, Missouri, USA). Each drug was prepared at a concentration of 10 mg/mL in sterile distilled water, except RIF, which was dissolved in dimethylformamide (DMF). Stock solutions were filter-sterilized and stored at −20°C for not more than one month.

### Media

Conventional LJ medium was prepared as des-cribed by Canetti *et al.* (23). The LJ medium for NRA was prepared with a slight modification: 1 mg/mL KNO_3_ was added to the LJ medium, with or without antibiotics, dissolved by stirring and then aliquoted and inspissated once for 50 minutes at 80°C. For NRA, the bottles containing LJ with antibiotics and KNO_3_ were used in duplicates while control tubes were used in triplicate.

#### Griess reagent

Fifty percent (vol/vol) concentrated hydrochloric acid (HCl), 0.2% (wt/vol) sulphanilamide, and 0.1% (wt/vol) n-1-naphthylethylenediamine dihydrochloride were prepared in small volumes and were mixed shortly before use in the ratio: 1 part: 2 part: 2 part respectively.

#### DST by PM

The PM was carried out on the LJ medium according to the laboratory's standard procedures with the recommended critical concentrations of 40 µg/mL for RIF, 0.2 µg/mL for INH, 2 µg/mL for EMB, and 4 µg/mL for STR (23, 24).

#### DST by NRA

NRA was performed according to the method described by Angeby *et al.* (14). Bacterial suspensions were made from a sub-cultured LJ tube by dispensing two 1-µL loopfuls of bacteria in 0.5 mL of phosphate-buffered saline (PBS) (pH 7.4) in 7.5-mL screw-cap bottles containing a few 3-mm diameter glass beads and vortexed to obtain a uniform solution. To obtain the turbidity of McFarland standard no. 1, approximately 2.5 mL of PBS was added. Part of the suspension was diluted at 1:10 in PBS. For each strain, 0.2 mL of the undiluted suspension was inoculated into tubes containing the LJ medium with KNO_3_ and the antibiotics in duplicates to reduce the risk of contamination and to assess the reproducibility of the test while 0.2 mL of the 1:10 dilution was inoculated into three drug-free tubes containing LJ with KNO_3_. The latter tubes served as growth controls. The tubes were incubated at 37 °C. After seven days, 0.5 mL of Griess reagent was added to one drug-free control tube. If any colour change could be observed, the corresponding antibiotic-containing tubes were also tested, and susceptibility results were read. If no colour change was observed in the growth control tube, this tube was discarded, and the other two control tubes and the antibiotic-containing tubes were re-incubated. The procedure was then repeated at day 10, using the second growth control, and if needed, also at day 14, using the last growth control tube.

The results were classified as negative if no colour change of the medium was observed and positive if pink to violet colour appeared in the medium. An isolate was considered to be resistant to a certain drug if there was a colour change in the antibiotic-containing tube in question greater than that in the 1:10 diluted growth control on the same day.

### Analysis of data

The MedCalc software (MedCalc, Belgium) wasused for calculating the statistical parameters, such as sensitivity, specificity, and accuracy. The predictive values were calculated using the prevalence of RIF, INH, STR, and EMB resistance in all the TB cases in Varanasi, India. The agreement between the NRA and the standard PM was determined by the κ-statistic. The κ value, a measure of test reliability, was interpreted as follows: <0.2 poor; 0.21-0.4 fair; 0.41-0.6 moderate; 0.61-0.8 good; and ≥0.81 excellent (25).

## RESULTS

DST for the first-line antitubercular drugs, i.e. RIF, INH, STR, and EMB, was performed with 286 clinical MTB isolates. The results of 107, 138, and 41 isolates were obtained after 7, 10, and 14 days respectively and were compared with those produced by the gold standard PM. The results are shown in the Table. For RIF, 123 and 161 strains were detected to be resistant and susceptible respectively by means of both the methods while two false-negative results were produced with the NRA. For INH, both the methods detected 126 resistant strains and 156 susceptible strains; four false-negative results were obtained with the NRA. For EMB, 115 and 162 strains were identified as true-positive and true-negative respectively by both the methods while 7 and 2 strains were misidentified as susceptible and resistant respectively by NRA. For STR, 139 isolates were correctly detected as resistant and 121 as sensitive by both the methods; 26 isolates gave discrepant results, 18 of which were false-negative **i.e.** resistant by PM while being susceptible by NRA, and 8 isolates were false-positive **i.e.** susceptible by PM but resistant by NRA. An excellent agreement was found between the two tests with the κ values of 0.98, 0.97, 0.81, and 0.93 for RIF, INH, STR, and EMB respectively. The sensitivity of the NRAcompared to that of PM was observed to be 98.4%, 97%, 88.5%, and 94.2% for RIF, INH, STR, and EMB respectively. Specificities were 100%, 100%, 94%, and 99% for RIF, INH, STR, and EMB respectively. The positive predictive values were 100%, 100%, 95%, and 98% for RIF, INH, STR, and EMB respectively. The negative values were 99%, 98%, 87%, and 96% for RIF, INH, STR, and EMB respectively. The accuracy of NRA was high with the PM for RIF (99.2%), INH (98.5%), and EMB (96.6%), except for STR (91%). An excellent agreement was observed in the results for RIF and INH. A good correlation was also found for EMB whereas some problems existed in detecting STR resistance and susceptibility (Table).

**Table.** Comparison of results of drug-susceptibility testing of 286 strains of *Mycobacterium tuberculosis* by PM and NRA

Antitubercular drugs	PM	No.		Nitrate reductase assay				
		Resistant strains	Sensitive strains	Sensitivity (%)	Specificity (%)	Positive predictive value (%)	Negative predictive value (%)	Accuracy (%)
Rifampicin	Resistant	123	2	98.4	100	100	99	99.2
	Susceptible	0	161					
Isoniazid	Resistant	126	4	97	100	100	98	98.5
	Susceptible	0	156					
Streptomycin	Resistant	139	18	88.5	94	95	87	91
	Susceptible	8	121					
Ethambutol	Resistant	115	7	94.2	99	98	96	96.6
	Susceptible	2	162					

NRA=Nitrate reductase assay; PM=Proportion method

## DISCUSSION

The most worrisome trend during recent years is an increase in multidrug-resistant (i.e. resistant to RIF and INH) TB strains. Rapid detection of MDR strains is very important to restrict their spread in the population. Current methods for DST of MTB are either costly or very slow. So, a cost-effective and rapid drug-susceptibility method is required to guide the treatment of TB. Results of meta-analysis of Martin *et al.* about NRA suggest that the NRA is highly sensitive and specific for determining RIF- and INH-resistant TB in both culture isolates and directly on clinical sputum specimens (26). Most studies had a sensitivity of 95% or greater, and nearly all were 100% specific with high degree of accuracy. The average TAT was 5-12 days with indirect NRA and 14-21 days with direct NRA. The biggest asset of NRA is that there is no need to change the laboratory infrastructure as it is performed in the classical LJ medium, routinely used in TB laboratories, with the addition of KNO_3_. There is no need of any sophisticated equipment or expensive reagent, making it a widely-used method. Results are easy to observe by a colour change of the medium.

In the present study, an excellent agreement between the results of NRA and PM was observed with the κ values of 0.98, 0.97, 0.81, and 0.93 for RIF, INH, STR, and EMB respectively. RIF together with INH are the most important drugs for the treatment of TB. Sensitivities of NRA for RIF and INH were 98.4% and 97% respectively whereas specificity was 100% for both the drugs. However, an acceptable result of NRA was also found for EMB, with sensitivity and specificity of 94.2% and 99% respectively but some problems existed in detecting STR resistance. Other studies have also reported high sensitivity and specificity values for INH and RIF (14, 27).

The accuracy with the PM was high for all the drugs used in this study, except STR which does not come under the standard level, according to the criteria established by the WHO/IUATLD Supranational Laboratory Network, which has proposed the accuracy levels of 99.0% and 97.0% for RIF and INH respectively and 92.0% for EMB and STR as reasonable performance goals for reference laboratories. Although it has been already reported that STR is a difficult drug to test even by standard methods (28), the performance of NRA for STR was not acceptable because the expected level of agreement (92%) was not achieved. However, the κ value of the tests for STR showed that there is an excellent agreement (0.81) between the two phenotypic tests.

The performance of NRA was evaluated in a multicentre study by Martin *et al.* to ascertain the susceptibility of MTB against first-line antitubercular drugs (29). The accuracy was greater than 97% for INH, EMB, and RIF while that for STR was inferior (85.3%). In another study, Martin *et al.* reported the evaluation of NRA for ofloxacin, a second-line drug, and found complete agreement with the agar PM (30). Therefore, NRA has the capability to be used also for the evaluation of second-line drugs. In addition, Lemus *et al.* evaluated indirect NRA with 320 strains of MTB and found an overall agreement of 98.8% between the NRA and the PM (31).

Recently, Khan and Sarkar have developed a dormant stage-specific anti-TB screening protocol in microplate format using Wayne's hypoxic model and nitrate reductase activity in *M. bovis* BCG (Bacillus Calmette-Guérin) culture (32). They concluded that the assay provides an easy, inexpensive, rapid, robust and high-content screening tool to search novel anti-TB molecules against both active and dormant bacilli with signal to noise ratio and Z factor, i.e. 8.5 and 0.81 respectively.

With the objective of reducing the TAT of DST by omitting the pre-isolation step, the NRA has been applied directly on clinical sputum specimens. A full agreement was observed for the detection of RIF resistance while some discordant results were obtained for other drugs (33). Visalakshi *et al.* observed sensitivity and specificity of the direct NRA and indirect PM to be 94% and 98%, and 100% and 98% for RIF and INH respectively with an excellent agreement between the two tests (21). Moreover, Shikama *et al.* stated 100% sensitivity and specificity of NRA for RIF (34). In another study of Shikama *et al.*, reproducibility of NRA was 100% for INH and EMB and 97% for STR and RIF, with 98.3% agreement between the results of NRA and PM for INH and RIF (35).

Syre *et al.* used colorimetric nitrate reductase-based antibiotic-susceptibility (CONRAS) test for DST of MTB against INH and RIF in liquid cultures (36). The results were produced within five days, indicating that the CONRAS test is an alternative in all settings, particularly for resource-poor countries.

Being advantageous, NRA has some limitations, such as some strains (<1%) of MTB lack nitrate reductase (37) rendering the test invalid. However, in our setting, we could not find any such strain in routine biochemical tests, including nitrate reductase test. In addition, nitrate might be reduced to nitric oxide beyond nitrite which cannot be detected by Griess reagent. Hence, zinc-dust was added to all negative tubes (22). Zinc reduces nitrate rapidly, and a true-negative test will directly turn red while there will be no change in colour in a tube where reduction has passed beyond nitrite. Another possible limitation of NRA is that it can give positive results with atypical mycobacteria *M. kansasii, M. szulgai, M. flavescens, M. terrae* complex, and some rapid growers (37) while *M. bovis* is nitrate-negative.

### Conclusions

Our results indicate that NRA is suitable for the early determination of INH and RIF resistance. On the basis of the findings, we conclude that NRA has the potential to be a useful tool for rapid DST of MTB in resource-poor countries with limited laboratory fa-cilities because of its low-cost, rapidness, reproducibility of results, simplicity and lack of requirement of expensive reagents and equipment.

## ACKNOWLEDGEMENTS

The authors thank Dr. Juan Carlos Palomino for providing the Medcalc software through e-mail. The authors are also thankful to Dr. VL Nag and Dr. G Nataraj for reference strains.
